# CANOMAD unmasked by COVID‐19 in a man with Waldenström's macroglobulinaemia

**DOI:** 10.1002/jha2.210

**Published:** 2021-08-12

**Authors:** Hamish D. Morrison, Jonathan Cleaver, Natasha Lander, Philippa Lowden, Kate Hale, Kanchan Sharma, James Stevens

**Affiliations:** ^1^ Neurology Department North Bristol NHS Trust Bristol UK; ^2^ Population Health Sciences University of Bristol Bristol UK

**Keywords:** cold agglutinin, immunoglobulin, neuropathy, WM

## 1 INTRODUCTION

CANOMAD is a rare syndrome encompassing chronic ataxic neuropathy, ophthalmoplegia, IgM paraprotein, cold agglutinins and disialosyl antibodies [1, 2]. We present a case of acute craniobulbar palsy associated with COVID‐19 in which the combination of an established IgM paraprotein and the later emergence of a typical and persistent ganglioside antibody profile ultimately revealed a diagnosis of CANOMAD syndrome. We review the clinical presentation, diagnostic work‐up and current treatment.

## 2 CASE PRESENTATION

A 61‐year‐old right‐handed male presented with a 24‐h history of altered speech, diplopia, facial weakness and dysphagia preceded by 4 days of symptoms consistent with COVID‐19. His background included a 3‐year history of Waldenström's macroglobulinaemia (WM)—MYD88 positive, IgM kappa paraproteinaemia 4g/L—and a sensory peripheral neuropathy; presumed related to the monoclonal gammopathy. His presenting neuropathy included subjectively reduced sensation in a stocking distribution bilaterally and mild gait ataxia. Examination findings at the point of diagnosis included global areflexia, impaired vibration sense throughout the lower limbs with difficulty in tandem walking and preserved limb power. He was able to walk unaided with an unlimited exercise tolerance. Nerve conduction studies revealed evidence of a large fibre sensory and motor peripheral neuropathy, with mild slowing and prolonged distal motor latencies but without reaching electrodiagnostic criteria for demyelination. He received four cycles of rituximab at diagnosis of WM for the neuropathy and remained stable for over 2 years. However, in the preceding 5 months to admission he reported progressive symptoms of gait ataxia and new reduced grip strength.

Admission vital signs were stable. Cranial nerve examination revealed a right‐sided ptosis, complex ophthalmoplegia, facial diplegia, poor palatal lift, tongue weakness and dysarthria. Limb examination demonstrated widespread mild proximal weakness, areflexia, impaired vibration throughout both legs and proprioception loss to the ankles. Nasopharyngeal swab subsequently confirmed SARS‐CoV‐2 infection by RT‐PCR, although chest X‐ray was normal. Blood tests revealed a lymphopaenia (0.96 × 10^9^ L), C‐reactive protein of 9 mg/ml and normal renal function. Cold agglutinins were identified and total IgM level was 7.51 g/L (0.35–2.42). Further blood tests were unremarkable, including HbA1C, plasma viscosity, creatine kinase, vitamin B12, folate, thyroid stimulating hormone, anti‐nuclear antibodies, complements, anti‐neutrophil cytoplasmic antibodies, hepatitis B and C serology, HIV, acetylcholine receptor antibodies and anti‐myelin associated glycoprotein (MAG) antibodies. Magnetic resonance imaging (MRI) of the brain was normal. Cerebrospinal fluid analysis revealed a mildly elevated protein of 0.84 g/L (<0.4) but no organism, cells or abnormal findings on cytology.

Twenty‐four hours later, the patient's respiratory condition deteriorated with radiological evidence of airspace consolidation. Dexamethasone and Remdesivir were commenced for severe COVID‐19 and he required noninvasive ventilation. Neurophysiological studies demonstrated evidence of demyelination with motor conduction block, prolonged distal motor latencies and reduced conduction velocities. Intravenous immunoglobulin (IVIg), 0.4 g/kg/day for 5 days, was administered for a presumptive diagnosis of Guillain–Barré syndrome (GBS). One week following IVIg treatment, there was complete resolution of ophthalmoparesis, facial diplegia and limb weakness with improving bulbar function. Eleven days into admission, respiratory function had normalised and the patient was discharged home.

**FIGURE 1:**
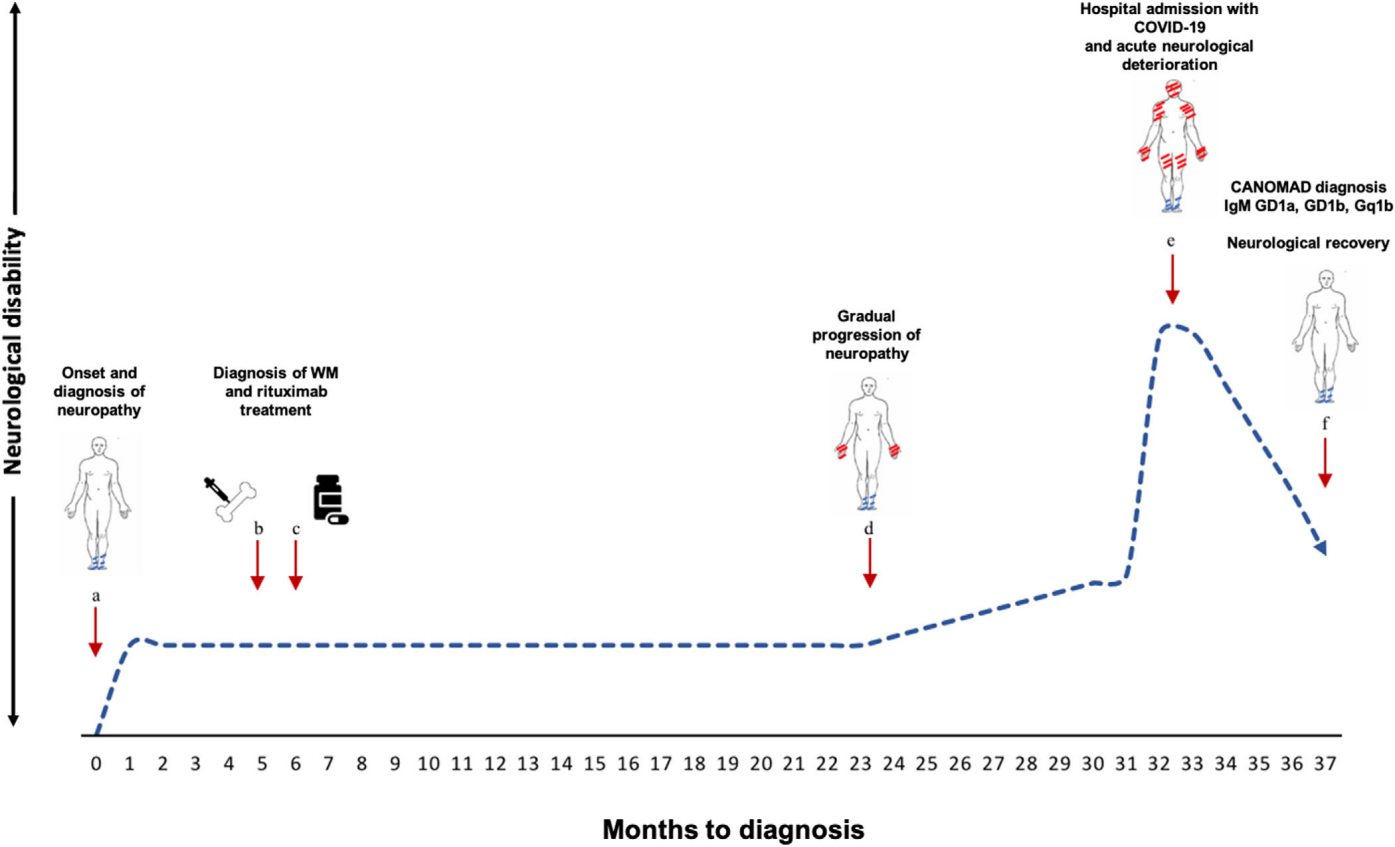
Clinical event timeline and estimation of functional neurological disability (neurological disability based on authors interpretation of subjective and objective markers of neurological function throughout disease course from presentation). Onset and diagnosis of neuropathy with sensory symptoms predominantly affecting the feet (a); bone marrow biopsy confirming WM (b), with rituximab treatment given 1 month later (c); progressive deterioration of sensory symptoms in feet, unsteady gait and weakness of grip strength (d); hospital admission with COVID‐19 and craniobulbar relapse treated with IVIg (e); diagnosis of CANOMAD and follow‐up demonstrating resolution of craniobulbar symptoms, marked improvement in motor function and mild residual sensory ataxia (f). Extent of sensory and motor involvement shown in blue and red, respectively

Pre‐IVIg anti‐ganglioside antibody panel subsequently demonstrated IgM titres of GD1a, GD1b and Gq1b all >1000 (<500) persisting at 2‐month follow‐up, corroborating a unifying diagnosis of CANOMAD. Repeat examination revealed a persistent sensory ataxia, but mobility had returned to baseline level. Figure 1 illustrates the clinical trajectory.

## 3 DISCUSSION

CANOMAD is characterised by a chronic neuropathy with sensory ataxia and variable motor weakness, typically involving the ocular and bulbar muscles, occurring in the presence of monoclonal IgM directed against disialosyl ganglioside epitopes (GD1b, GD3, GT1b and GQ1b). Overt haematological malignancy is seen in around one‐third. The associated neuropathy is often demyelinating, although not all neurophysiological testing will reveal this, such as in our case on initial testing. Ataxic neuropathy is frequently the dominant symptom, potentially causing severe disability. Disease trajectory is variable, typically extending over decades, with progression in two‐thirds and a third relapsing‐remitting [3]. Misdiagnosis is common, in part because fewer than half of the patients exhibit the full spectrum of clinical features, ultimately delaying appropriate treatment. Craniobulbar manifestations may be intermittent or persistent and often herald relapse, raising suspicion for CANOMAD in patients with an IgM paraprotein and undifferentiated neuropathy.

Intercurrent infection is recognised as a trigger for CANOMAD relapse, although there remains limited published data to substantiate this [1]. Hitherto, this is the first reported case of CANOMAD relapse associated with COVID‐19. Immune‐mediated neuropathy and exacerbations of chronic neuromuscular disease have been described amongst possible complications of COVID‐19 [4, 5]. However, other evidence implies a lack of causality, for example, between COVID‐19 and GBS [6]. SARS‐CoV‐2 has the ability to bind to sialic acid containing glycoproteins and gangliosides often found on peripheral nerves [7]. Shared glycoprotein epitopes may have incited a high degree of specific immune stimulation, triggering the observed CANOMAD relapse. Positive GD1b antibodies in a Miller Fisher syndrome patient with COVID‐19 may lend support to this theory of immune cross‐reactivity between the SARS‐CoV‐2 spike and peripheral nerve gangliosides [8]. However, the specificity of this proposed immune response remains unproven.

Neuropathy is present in up to 31% of patients with IgM monoclonal gammopathy and as many as 62.5% of patients with WM [9, 10]. As neuropathy may be an indication for treatment in WM, identification of the underlying aetiology is important. The International Workshops on WM (IWWM) published recommendations in 2017 including a schematic decision tree for evaluating IgM‐associated peripheral neuropathies [11]. However, a rigid interpretation of this pathway would only result in testing for ganglioside antibodies alongside anti‐MAG antibodies in patients demonstrating conduction slowing/demyelination on electrophysiological testing. Findings from a recent large study of 45 patients with CANOMAD revealed a demyelinating pattern in 60%, with predominantly axonal features in 27% [3]. Therefore, it is important not to rely solely on the reported pattern of electrophysiological abnormality, and maintain a high index of suspicion in cases exhibiting the aforementioned neurological phenotype.

There remains a lack of evidence‐base for the treatment of CANOMAD. IVIg and rituximab are reportedly most efficacious, whereas corticosteroids and other forms of immunosuppression have little or unfavourable impact [3]. In this case, rituximab—administered for the haematological malignancy and associated neuropathy—likely had a stabilising effect on the neuropathy, in keeping with previously described cases [3, 12].

## 4 CONCLUSION

Whilst the CANOMAD acronym may prove a useful aide memoir, not all cases exhibit the full spectrum of clinical features. Craniobulbar relapses are common and may be precipitated by infection, including COVID‐19. Algorithmic approaches may mislead the diagnostic process, and high degree of clinical vigilance is required, with screening for disialosyl antibodies considered in those with chronic ataxic neuropathy, IgM paraproteinaemia and negative anti‐MAG antibodies. Closer collaboration between haematologists and neurologists is encouraged to facilitate timely diagnosis and improve clinical outcome.

## AUTHOR CONTRIBUTIONS

Hamish D. Morrison and Jonathan Cleaver conceptualised the manuscript. Hamish D. Morrison, Jonathan Cleaver, Natasha Lander and Philippa Lowden prepared the first draft. Hamish D. Morrison, Jonathan Cleaver, Kate Hale, Kanchan Sharma and James Stevens revised the manuscript.

## Data Availability

Not applicable to this article as no datasets were generated or analysed during the current study.
